# Supplementary material for: Modelling Skylarks (Alauda arvensis) to Predict Impacts of Changes in Land Management and Policy: Development and Testing of an Agent-Based Model
Source: PLoS One. 2013 Jun 6;8(6):e65803. doi: 10.1371/journal.pone.0065803 (PMC3675089; doi:10.1371/journal.pone.0065803)
Supplement: Supporting Information S4 — The skylark ODdox as a zipped archive. (ZIP) [file pone.0065803.s004.zip › Skylark_ODdox/structrectangle.html]

ALMaSS Skylark ODdox: rectangle Struct Reference


ALMaSS Skylark ODdox
 2.0


- Main Page
- Related Pages
- Classes
- Files

- Class List
- Class Index
- Class Hierarchy
- Class Members

Public Attributes

rectangle Struct Reference

A struct defining two x,y coordinate sets of positive co-ords only.
More...

`#include <populationmanager.h>`

List of all members.

|  |  |
| --- | --- |
| Public Attributes | |
| unsigned | m\_x1 |
| unsigned | m\_x2 |
| unsigned | m\_y1 |
| unsigned | m\_y2 |

---

## Detailed Description

A struct defining two x,y coordinate sets of positive co-ords only.

---

## Member Data Documentation

unsigned rectangle::m\_x1

Referenced by Skylark\_Base::InSquare(), TSkylarkList< T >::Probe(), and Population\_Manager::Probe().

unsigned rectangle::m\_x2

Referenced by Skylark\_Base::InSquare(), TSkylarkList< T >::Probe(), and Population\_Manager::Probe().

unsigned rectangle::m\_y1

Referenced by Skylark\_Base::InSquare(), TSkylarkList< T >::Probe(), and Population\_Manager::Probe().

unsigned rectangle::m\_y2

Referenced by Skylark\_Base::InSquare(), TSkylarkList< T >::Probe(), and Population\_Manager::Probe().

---

The documentation for this struct was generated from the following file:

- populationmanager.h


- rectangle
- Generated on Thu Jan 10 2013 13:15:37 for ALMaSS Skylark ODdox by
   1.8.1.1
